# Developmental Population Pharmacokinetics and Dosing Optimization of Cefepime in Neonates and Young Infants

**DOI:** 10.3389/fphar.2020.00014

**Published:** 2020-02-04

**Authors:** Yang Zhao, Bu-Fan Yao, Chen Kou, Hai-Yan Xu, Bo-Hao Tang, Yue-E Wu, Guo-Xiang Hao, Xin-Ping Zhang, Wei Zhao

**Affiliations:** ^1^School of Medicine and Health Management, Tongji Medical College, Huazhong University of Science and Technology, Wuhan, China; ^2^Department of Clinical Pharmacy, School of Pharmaceutical Sciences, Shandong University, Jinan, China; ^3^Department of Neonatology, Beijing Obstetrics and Gynecology Hospital, Capital Medical University, Beijing, China; ^4^Department of Neonatology, Shandong Provincial Qianfoshan Hospital, The First Affiliated Hospital of Shandong First Medical University, Jinan, China; ^5^Department of Pharmacy, Shandong Provincial Qianfoshan Hospital, The First Affiliated Hospital of Shandong First Medical University, Jinan, China

**Keywords:** cefepime, pharmacokinetics, infections, neonates, infants

## Abstract

**Objective:**

Cefepime is used to treat severe infections in neonates. Pharmacokinetic data have only been evaluated among preterm neonates and population pharmacokinetic model lacked external validation. Hence, our aim is to obtain the population pharmacokinetic parameters of cefepime with large sampling and optimize the cefepime dosage regimen for neonatal infection based on developmental pharmacokinetics–pharmacodynamics.

**Methods:**

Blood samples from neonates and young infants treated with cefepime were collected using the opportunistic sampling design. The concentration of cefepime was determined using high performance liquid chromatography with ultraviolet detection. The population pharmacokinetic model was established using NONMEM software.

**Results:**

One hundred blood samples from eighty-five neonates were analyzed. The population pharmacokinetics of cefepime were described by a one-compartment model with first-order elimination. Covariate analysis indicated that serum creatinine concentration, postmenstrual age and current weight had significant impact on the pharmacokinetic parameters of cefepime. Monte Carlo simulation results showed that the current dosage regimen (30 mg/kg, q12 h) had a high risk of insufficient dose. For 70% of neonates to obtain a higher free drug concentration than the minimum inhibitory concentration during 70% of the dosing interval, 50 mg/kg q12 h was needed with a susceptibility breakpoint of 4 mg/l. For a minimum inhibitory concentration of 8 mg/l, 40 mg/kg q8 h was recommended for all neonates.

**Conclusion:**

A population pharmacokinetic model of cefepime in neonates and young infants was established. According to simulation results based on the developmental pharmacokinetics–pharmacodynamics, different dosage regimens should be given depending on pathogens and the postmenstrual age.

## Introduction

Cefepime is the fourth-generation cephalosporin and is used to empirically treat severe nosocomial infections including pneumonia and meningitis in neonates (Capparelli et al., 2005; Pacifici and Marchini, 2017). It is against both susceptible gram-negative pathogens (such as *Enterobacter* and *Pseudomonas aeruginosa*) and susceptible gram-positive pathogens (such as *Streptococcus pneumoniae*). It plays an essential role in treating infections caused by *P. aeruginosa*, which develops resistance to third generation cephalosporins (Pacifici and Marchini, 2017).

Cefepime has wide distribution into body tissues and fluids, low plasma protein binding (≤20%) and it is primarily excreted unchanged by the kidneys (Pacifici and Marchini, 2017). Therefore, the function and maturation of the kidneys affects cefepime performance; the pharmacokinetics of neonates differ from those of adults and older children (Reed et al., 1997; Blumer et al., 2001). To the best of our knowledge, pharmacokinetic studies of cefepime has only been conducted in preterm neonates with narrow age range and the developed population pharmacokinetic models had not been externally validated (Capparelli et al., 2005; Lima-Rogel et al., 2008; Shoji et al., 2016). In addition, the developmental pharmacokinetic-pharmacodynamic based dosing recommendation of cefepime was not available in China. Obviously, the lack of these evidence-based data results in its use in an off-label manner in neonatal clinical practice, which could either increase the risk of drug-related toxicity or encourage the spread of clinical antibiotic resistance.

We therefore aimed to use patients including both preterm and term neonates as our study subjects to determine the population pharmacokinetic parameters of neonates and determine an evidence-based therapeutic dose regimen based on pharmacokinetic-pharmacodynamic simulation to improve cefepime therapy in infection treatment in Chinese neonates and young infants.

## Method

### Study Design

This trial was a prospective, open label pharmacokinetic study of cefepime, performed at the Beijing Obstetrics and Gynecology Hospital and Shandong Provincial Qianfoshan Hospital. The inclusion criteria were: neonates and young infants with postmenstrual age (PMA) less than 48 weeks, neonatal patients were treated regularly with cefepime and written parental consent to participate the study was provided. Exclusion criteria were: the patients used other antibiotics, expected survival time was less than the treatment cycle and other reasons that the researcher determined the patient to be unsuitable for inclusion. This study was approved by the ethics committee of Shandong Provincial Qianfoshan Hospital.

### Dosing Regimen and Pharmacokinetic Sampling

Cefepime (Maxipime, Sino-American Shanghai Squibb Pharmaceuticals Ltd, Shanghai, China), 30 mg/kg q12 h, was administered intravenously. All samples were collected with an opportunistic sampling design (Leroux et al., 2015). Blood volume of each sample was 0.2 ml and infusion and sample times were precisely recorded. A further sample was extracted from the remaining blood after routine biochemical tests. Samples were only included once they had validated sampling information. Blood samples were centrifuged (4,000 rpm for 10 min) and plasma samples were stored at −80°C.

### Cefepime Analysis

The cefepime blood concentration was determined using high-performance liquid chromatography with ultraviolet detector taking cefotiam as the internal standard. The range of calibration curve was 0.2–200 μg/ml and the lower limit of quantification (LOQ) was 0.2 μg/ml. The interday and intraday coefficients of variation were less than 6.3 and 11.9%, respectively.

### Population Pharmacokinetic Modeling of Cefepime

Pharmacokinetic analysis was performed on the nonlinear mixed effects modeling program NONMEM V 7.2 (Icon Development Solutions, USA). In order to estimate pharmacokinetic parameters and their variability, first order conditional estimation (FOCE) method with interaction was used in our study. Covariate analysis followed two steps including forward (p < 0.05) and backward (p < 0.01) selection process. The likelihood ratio test was performed, with which the effect of each variable on model parameters could be tested. We investigated the effects of birth weight, current weight, gestational age, postnatal age, postmenstrual age and serum creatinine concentration (collected within ≤48 h of pharmacokinetic sampling) which may affect pharmacokinetic parameters potentially. The performance of model was validated by graphical and statistical criteria, including Goodness-of-fit plots, bootstrap and normalized prediction distribution errors (NPDE). Both internal validation and external validation were applied to confirm the predictive performance of the model. The detailed technical information of model building, covariate analysis and model validation methods are available in **Supplemental File 1**.

### Dosing Regimen Evaluation and Optimization

Cefepime’s effect on bacteria is time-dependent because the pharmacokinetics-pharmacodynamics relationship is the duration of free antimicrobial drug concentration higher than minimum inhibitory concentration (fT > MIC). To get the maximal antibacterial activity, 70% of patients should reach the target (fT > MIC) during 70% of the dosing interval (Craig, 1998; Dudley and Ambrose, 2000). The proportion of free cefepime is approximately 80% (B-MS C, 2009; Pacifici and Marchini, 2017). According to EUCAST (European Committee on Antimicrobial Susceptibility Testing), for *P. aeruginosa* and *Enterobacter species*, cefepime’s MIC is 8 and 4 μg/ml, respectively (EUCAST, 2018). For *S. pneumoniae*, *Haemophilus influenzae* and other pathogens, cefepime’s MIC is less than 4 μg/ml. All pathogens mentioned above can lead to pneumonia or meningitis in neonates (Hooven and Polin, 2017). Therefore, MIC targets of 8 and 4 μg/ml were selected to optimize dosing regimen.

Monte Carlo simulation was performed using the parameters estimated in the final model. Cefepime dose was simulated on a milligram per kilogram basis, according to age group. First, the current treatment regimen (30 mg/kg, q12 h) in the original data set was simulated. Then, the original data set was simulated 100 times and the time during which the plasma concentration of every original neonate was above the MIC was calculated. If a treatment regimen was found that did not attain the target in more than 50% of patients, we considered increasing the dose and/or frequency and the optimal dosing regimens were given to virtual patients (Visser et al., 1993; Lan and Colford, 2000; Drusano, 2003). The target achievement probability of each regimen was calculated to select the optimal regimen.

## Results

### Study Population

Eighty-five neonates were initially enrolled from 2017 to 2018. All neonates met the inclusion and exclusion criteria and informed consent was obtained. The mean values of PMA and weight of the 85 patients were 39.2 weeks and 3,210 g, respectively. Patient characteristics are summarized in **Table 1**.

**Table 1:** Patient’ demographic characteristics in 85 neonates for model building.

	Number	Mean (SD)	Median (Range)
**Patients**	85		
Gestational age (weeks)		38.1 (2.80)	39.0 (28.0–41.6)
Postmenstrual age (weeks)		39.2 (3.35)	40.1 (30.6–45.1)
Postnatal age (days)		7.58 (3.83)	8(1–25)
Birth weight (g)		3092 (620)	3120 (980–4210)
Current weight (g)		3210 (678)	3353 (950–4350)
Serum creatinine concentration (µmol/l)		34.3 (17.1)	28.5 (11.5–92.4)
**Cefepime treatment**			
Dose (mg/dose)		106 (31.8)	100 (30–190)
Dose (mg/kg/dose)		33.3 (8.31)	29.7 (25.2–53.9)

### Model Building

**Figure 1:**
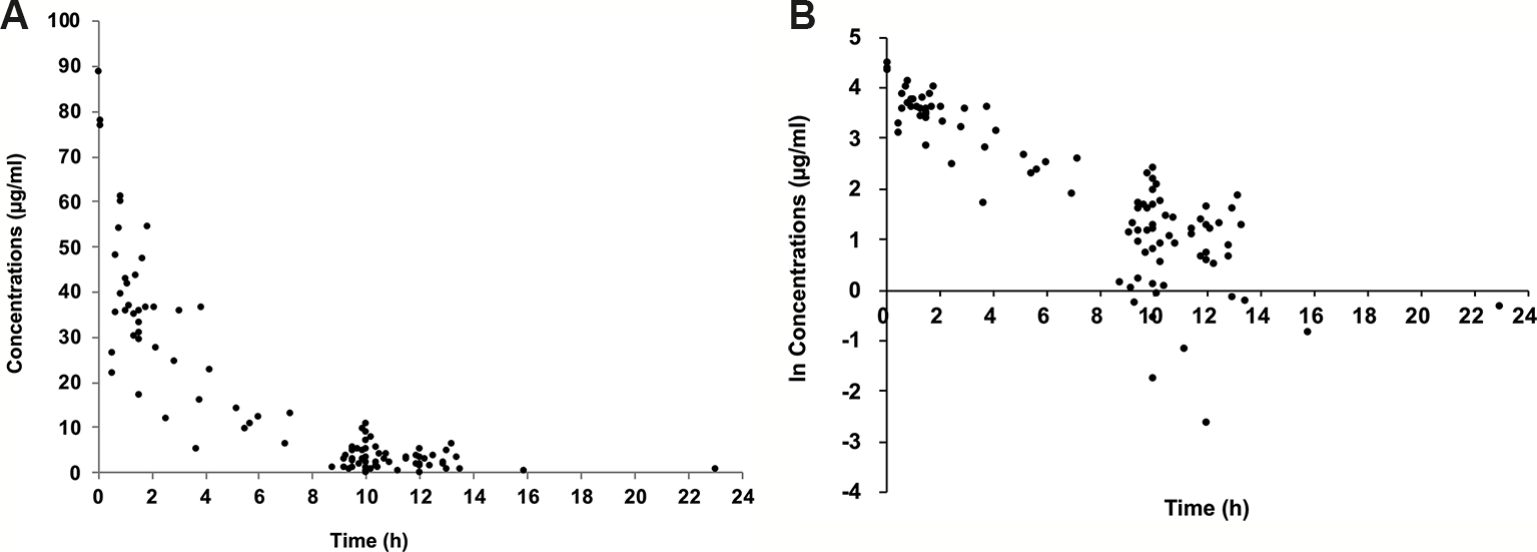
Plasma concentration of cefepime versus time since last dose. Concentration versus time curve **(A)**; ln concentration versus time curve **(B)**.

One hundred concentrations were available to build the population model. The concentrations of cefepime ranged from lower than the LOQ to 89.0 μg/ml. Three concentrations were lower than the LOQ, and each concentration was replaced by 0.1μg/ml which was half of LOQ. The concentration *versus* time curve and ln concentration versus time curve were shown in **Figures 1A, B**.

A one-compartment model with first-order elimination best describes the data. The pharmacokinetic parameters derived from the model included clearance (CL) and distribution volume (V) of cefepime. The exponential model best described the inter-individual variability. The variability was estimated for CL and V. Residual variability was best described using a proportional model.

### Covariate Analysis

The allometric size approach was used by incorporating *a priori* the current weight into the basic model (allometric coefficients of 0.75 for CL, 1 for V), which caused a significant drop in the objective function value (OFV) of 21.8 points. PMA was the most important covariate for CL and was related to a 7.5-unit drop in OFV. Another important covariate for CL was serum creatinine concentration, which reduced OFV by 6.4 units. A detailed covariate analysis process was shown in **Table 2**.

**Table 2:** Covariate analysis.

	PK Parameters	Objective Function Value
**Structural model**		***430.89***
**Allometric model**	**CL, V**	
***Current body weight***		***409.06***
**Impact of age**	**V**	
***Age***		*409.13*
**Impact of age**	**CL**	
***Age***		***401.58***
**Impacts of renal maturation and renal function**	**CL**	
***PMA and serum creatinine***		***395.15***

PMA, postmenstrual age. Bolded texts indicated a significant drop in the objective function value (OFV).

**Figure 2:**
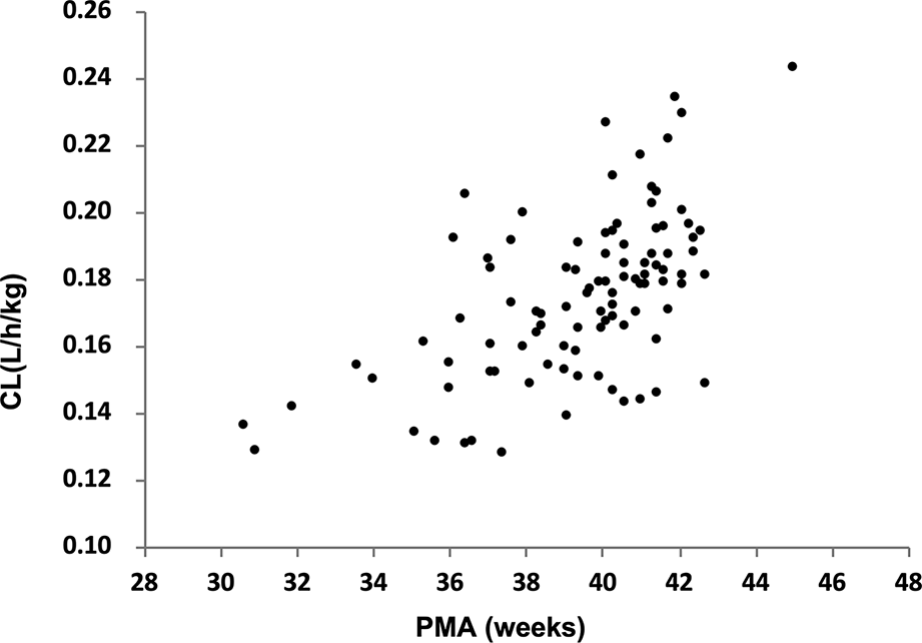
The relationship of cefepime weight-normalized CL (l/h/kg) with PMA.

The parameter estimated values of the final model were summarized in **Table 3**. The median (range) of estimated weight-normalized CL was 0.18 (0.13–0.24) l/h/kg and V at steady-state was 0.62 (0.38–0.85) l/kg. AUC_0–24_ at steady-state for the evaluated dosage regimen ranged from 112 to 379 mg*h/l. The clearance of cefepime increased with current weight and decreased with increased serum creatinine concentration in preterm and term neonates. The relationship of cefepime weight-normalized CL (L/h/kg) with PMA was shown in **Figure 2**.

**Table 3:** Population pharmacokinetic parameters of cefepime and bootstrap results.

Parameters	Full Dataset		Bootstrap	
	Final Estimate	RSE(%)	Median	5th–95th
V (L)				
V = θ1× (CW/3,352)				
θ1	2.07	8.40	2.06	1.79–2.46
CL(l/h)				
CL = θ2×(CW/3352)^0.75^ × F_age_ × RF				
θ2	0.589	6.20	0.586	0.530–0.649
F_age_ = (PMA/40)^θ3^				
θ3	1.16	49.5	1.21	0.283–2.042
RF = 1/(CREA/28.5)^θ4^				
θ4	0.218	45.4	0.238	0.068–0.363
Inter-individual variability (%)				
V	26.8	56.1	23.8	7.75–35.3
CL	15.3	87.6	15.5	3.88–24.2
Residual variability (%)	36.6	20.6	35.5	28.4–47.7

V, volume of distribution; CL, clearance; RF, renal function; CW, current weight in gram; CREA, serum creatinine concentration in μmol/l; PMA, postmenstrual age in weeks. In our population, 3,352 g, 40 weeks and 28.5 μmol/l are the median current weight (day of the study), postmenstrual age and serum creatinine concentration values, respectively.

### Model Evaluation

#### Internal Model Validation

**Figure 3:**
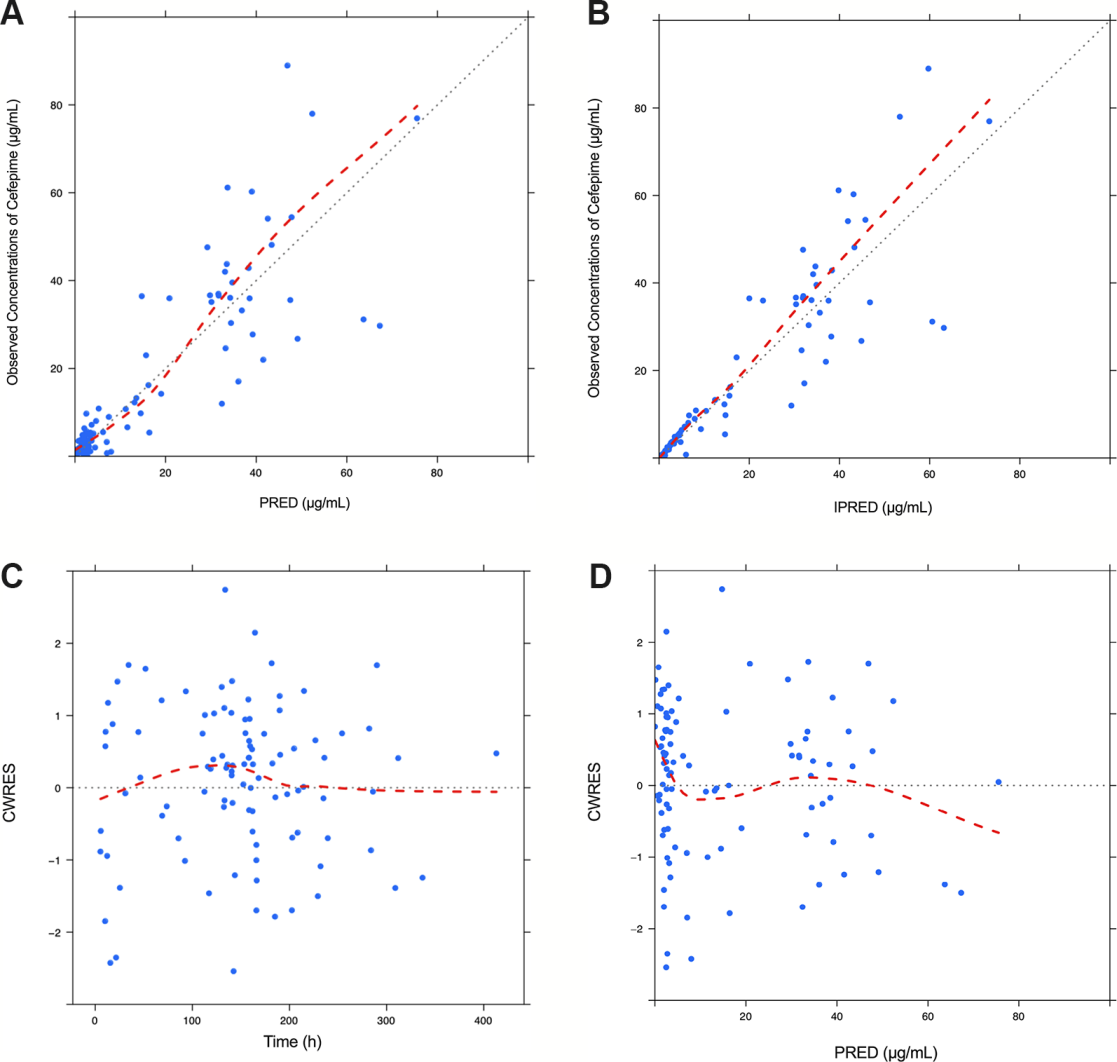
Diagnostic goodness-of-fit plots for the final population pharmacokinetic model of cefepime. Observed (DV) versus population prediction (PRED) **(A)**; DV versus individual prediction (IPRED) **(B)**; conditional weighted residuals (CWRES) versus time **(C)** and CWRES versus PRED **(D)**.

**Figure 4:**
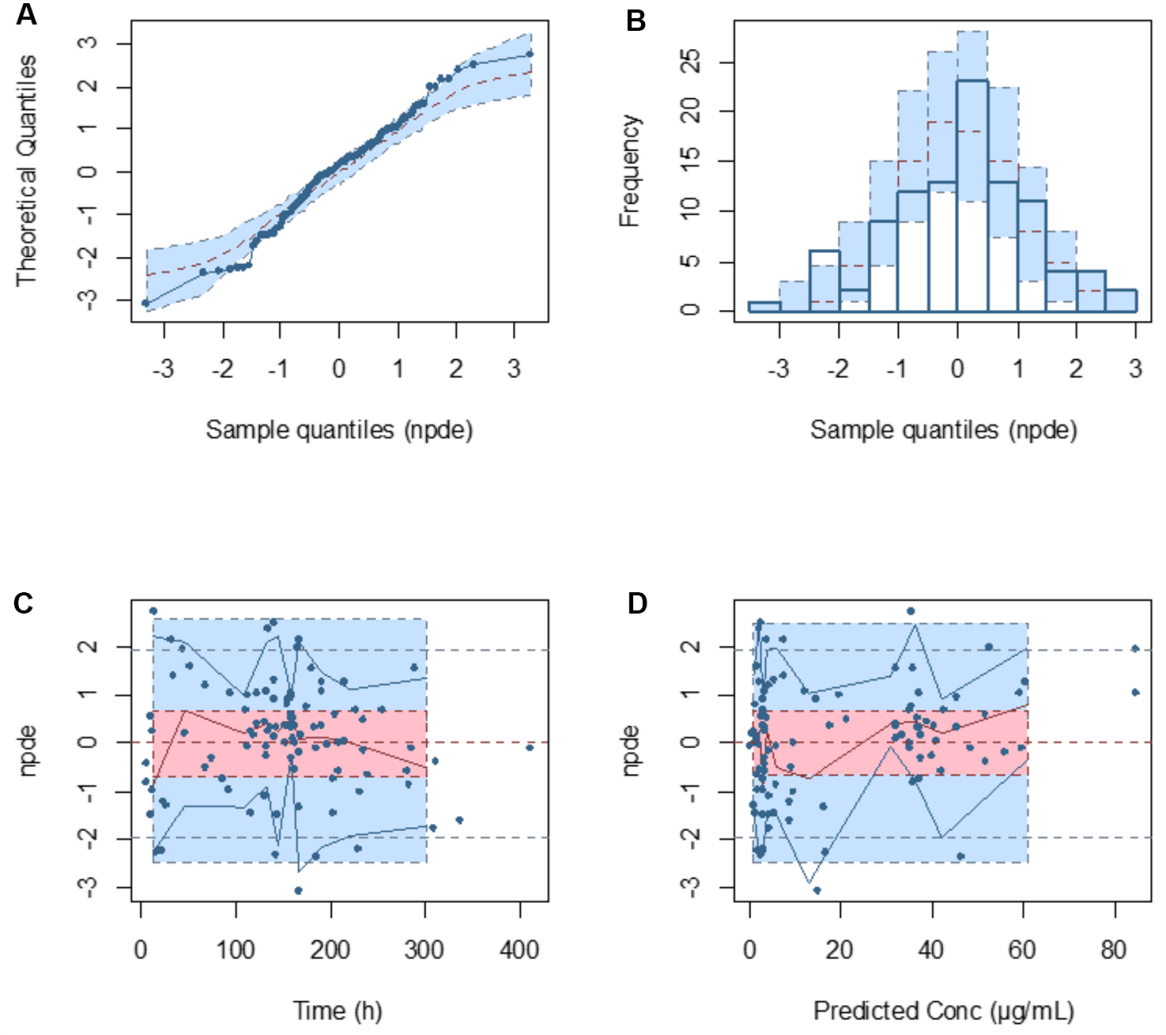
Normalized prediction distribution error (NPDE) metrics for the population pharmacokinetic model of cefepime. Normal Q–Q plot for NPDE **(A)**, distribution of NPDE **(B)**, and NPDE versus time after first dose **(C)** and versus predicted concentrations **(D)**.

Model diagnostics demonstrated acceptable goodness-of-fit for the final cefepime model. **Figures 3A, B** showed that there was no systematic bias on predictions. As shown in **Figures 3C, D**, no trends were found in the diagnostic plots of conditional weighted residuals (CWRES) *versus* time and population prediction (PRED). Moreover, the median parameter estimates obtained from the bootstrap program were consistent with the respective values from the final model, which indicates that the final model was stable and could be used to re-estimate population pharmacokinetic parameters (**Table 3**). The NPDE results were shown in **Figures 4A–D**. As shown in **Figures 4A, B**, NPDE distribution and histogram agreed well with the standard normal distribution and density, which indicates that the model fitted to the individual data well. The mean and variance of NPDE were 0.04 (Wilcoxon signed rank test p = 0.28) and 1.11 (Fisher variance test 0.41), respectively. As shown in **Figures 4C, D**, there was no trend in NPDE *versus* time and PRED.

#### External Model Validation

The external validation data from 15 patients were obtained using opportunistic pharmacokinetic sampling. All of them met the inclusion and exclusion criteria and the informed consent was obtained. The mean (SD; range) PMA and weight of the 15 neonates were 37.5 (5.1; 29.6–43.6) weeks and 2,602 (1,393; 750–4,900) g, respectively. Patient characteristics of external validation data were shown in **Table 4**. The mean prediction error (MPE) and mean absolute prediction (MAE) values were −8.3 and 11.1%, respectively. Ninety percent of patients were within the range of ±20% of MPE and all patients within ±30% of MPE.

**Table 4:** Patient demographic characteristics in 15 neonates for external validation.

	Number	Mean (SD)	Median (Range)
**Patients**	15		
Gestational age (weeks)		36.1 (4.7)	37 (29–42)
Postmenstrual age (weeks)		37.5 (5.1)	38 (29.6–43.6)
Postnatal age (days)		9.7 (6.6)	8 (3–25)
Current weight (g)		2602 (1393)	2560 (750–4,900)
Serum creatinine concentration (µmol/l)		42.5 (13.2)	45 (15–60)
**Cefepime treatment**			
Dose (mg/dose)		76.4 (39.5)	75 (25–140)
Dose (mg/kg/dose)		29.8 (1.5)	29.9 (28.6–33.3)

### Dosing Regimen Evaluation and Optimization

**Figure 5:**
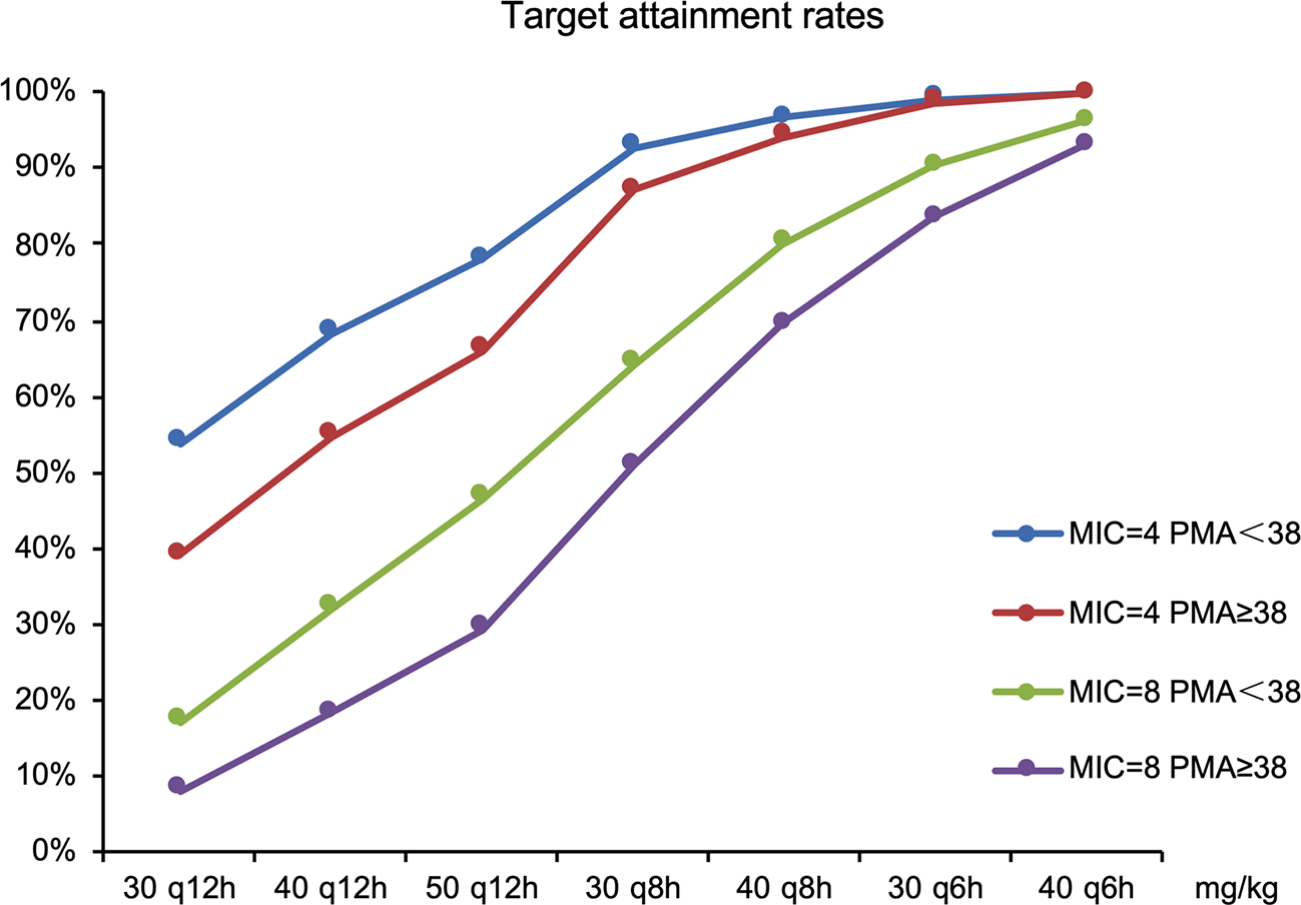
The target attainment rates as functions of simulated dose for standard MIC breakpoints.

The goal was for more than 70% of patients’ plasma concentration to be above the MIC during 70% of the dosing interval. The target attainment rates as functions of simulated dose for standard MIC breakpoints are shown in **Figure 5**.

Current dosage (30 mg/kg, q12 h) was far from the objective of drug efficacy. Therefore, dosages were adjusted; results included the following: 50 mg/kg q12 h was required for all neonates for an MIC of 4 mg/l and 78 and 66% of preterm (PMA <38 weeks) and term neonates (PMA ≥38 weeks), respectively, had plasma concentrations above the MIC. For MIC of 8 mg/l, 40mg/kg q8 h was recommended for preterm and term neonates with 80 and 70% of patients achieving the target (70% fT > MIC), respectively.

## Discussion

We developed a developmental pharmacokinetic-pharmacodynamic model to optimize dosing regimen of cefepime, in which we included a wider age group covering the whole neonatal population. The results showed that the one-compartment model with first-order elimination best fitted the population pharmacokinetics of cefepime.

In this study, the mean cefepime CL to neonates was 0.18 (0.13–0.24) l/h/kg. Previous reports showed that cefepime CL in neonates was approximately 0.07 to 0.20 l/h/kg (Capparelli et al., 2005; Lima-Rogel et al., 2008; Shoji et al., 2016). Thus, our results were consistent well with former research. Covariate analysis showed that creatinine clearance and PMA influenced CL the most. Cefepime was excreted primarily through the kidneys. Maturation of the kidney and whole body influenced cefepime clearance. Creatinine clearance reflects renal function, which is calculated from serum creatinine concentration. During the whole selection procedure, other factors did not affect pharmacokinetic parameters.

The duration of free cefepime plasma concentration remaining above MIC is an important indicator of cefepime’s effectiveness in fighting infections. Considering the conservative effectiveness of treatment and to decreasing drug resistance, our target was that 70% of neonatal patients had a cefepime concentration above the MIC for 70% of the dosing interval. EUCAST recommended PK-PD (Non-species related) cefepime breakpoints of 4 and 8 μg/ml for sensitive and resistant bacteria, respectively. As shown in the dosing regimen optimization section, the current dosing regimen (30 mg/kg, q12 h) was insufficient to treat neonatal infections. Therefore, our solutions were to increase dose every time or/and increase dosing frequency. With the principle of daily dosage minimum, some appropriate dosing regimens were suggested for different pathogenic bacteria species. All neonates needed 50 mg/kg q12 h when the susceptibility breakpoint was 4 mg/l. For MIC of 8 mg/l, 40 mg/kg q8 h was recommended for preterm and term neonates. Neonatal pneumonia and meningitis caused by *P. aeruginosa* could not be effectively treated with third-generation cephalosporin or regular cefepime treatment. It was therefore necessary to increase daily dosage; our results supported this presumption.

It is notable that we performed external validation in preterm and term neonates. On the one hand, it requires reliable models to accurately describe the original data set. On the other hand, pharmacokinetic–pharmacodynamic models must have good predictability to give a safe and effective evidence-based dosage regimen for preterm and term neonates. Therefore, external validation was an appropriate supplementary procedure to assess our model (Zhao et al., 2013). Finally, the model’s predictive performance was reliable for the predicting dosing regimen prediction.

However, this study had some limitations. Because our results were from a model-based simulation, the optimized dosing regimen should be practiced in clinical treatment and be supported by results from advanced clinical treatment.

## Conclusion

The population pharmacokinetics of cefepime was appraised through using large sampling through a wider age group covering the whole neonatal population. Current weight, PMA and serum creatinine concentration influenced cefepime clearance the most. External validation confirmed the reliability of this pharmacokinetic-pharmacodynamic model. Optimal dosing regimen, which was conducted according to pathogens and age group, was established based on developmental pharmacokinetics–pharmacodynamics.

## Data Availability Statement

All datasets generated for this study are included in the article/**Supplementary Material**.

## Ethics Statement

The studies involving human participants were reviewed and approved by Shandong Provincial Qianfoshan Hospital. Written informed consent to participate in this study was provided by the participants’ legal guardian/next of kin.

## Author Contributions

YZ and B-FY retrieved data, carried out the initial analyses and drafted the initial manuscript. CK, H-YX, B-HT, Y-EW and G-XH collected samples and recorded patient information. X-PZ and WZ conceptualized, designed and initiated the study. All the authors contributed to write the manuscript and approved the final manuscript as submitted.

## Funding

This study was supported by the National Science and Technology Major Projects for “Major New Drugs Innovation and Development” (2017ZX09304029-002), Young Taishan Scholars Program of Shandong Province and Qilu Young Scholars Program of Shandong University.

## Conflict of Interest

The authors declare that the research was conducted in the absence of any commercial or financial relationships that could be construed as a potential conflict of interest.
