# Supplementary material for: Developmental Population Pharmacokinetics and Dosing Optimization of Cefepime in Neonates and Young Infants
Source: Front Pharmacol. 2020 Feb 4;11:14. doi: 10.3389/fphar.2020.00014 (PMC7010644; doi:10.3389/fphar.2020.00014)
Supplement: Supplementary file 1 [file DataSheet_1.pdf]

***Population pharmacokinetic modeling of cefepime***

Inter-individual variability of the pharmacokinetic parameters was estimated using an exponential model and was expressed as following equation (Eqs. 1):

$\theta_i = \theta_{mean} * e^{\eta i}$ (1)

where $\theta_i$ represented the parameter value of the $i^{th}$ subject, $\theta_{mean}$ the typical value of the parameter in the population and ηi the variability between subjects which is assumed to follow a normal distribution with a mean of zero and variance ω2.

During forward selection process, a covariate was included if a significant ($p<0.05$, $\chi^2$ distribution with one degree of freedom) decrease (reduction>3.84) in the objective function value (OFV) from the basic model was obtained and a reduction in the variability of the pharmacokinetic parameter. Then, all the significant covariates were simultaneously added into a 'full' model. And then after that, each covariate was independently removed from the 'full' model. When the OFV increased more than 6.635 ($p<0.01$, $\chi^2$ distribution), the covariate was regarded as significantly correlated with the pharmacokinetic parameter and therefore it was retained in the final model.

***Model validation***

***(1) Internal model validation***

Goodness-of-fit plots, including observed (DV) *versus* population prediction (PRED); DV *versus* individual prediction (IPRED); conditional weighted residuals (CWRES) *versus* time and CWRES *versus* PRED were initially used to validate the model(1). A nonparametric bootstrap with re-sampling and replacement was conducted to assess the stability and performance of the final model. Re-sampling was repeated 1000 times and the values of parameters estimated from the bootstrap procedure were compared with those estimated from the original data set. The entire procedure was performed in an automated way, using PsN (v2.30)(2). The final model was also validated graphically and statistically using normalized prediction distribution errors (NPDE). One thousand datasets were simulated with the final population model parameters. NPDE results were provided graphically by the NPDE R package (v2.0)(3): (i) QQ-plot of the NPDE; (ii) histogram of the NPDE; (iii) NPDE *versus* Time after first dose; (iv) NPDE *versus* PRED. The NPDE is expected to follow the standard normal distribution.

***(2) External model validation***

In order to validate the model, the independent dataset was obtained and the same opportunistic PK sampling design was performed. Information of patients for external validation was available which included basic physiological information, dosing information, sampling information and covariate information.

Mean absolute prediction (MAE) and mean prediction error (MPE) were applied to calculate bias and imprecision of predictive performance (4) (Eqs. 2 and 3). In addition, the number of patients with MAE and MPE within the range of ±20 and ±30% were calculated (5).

$$MAE = \frac{1}{N} \sum \left| \frac{IPRED_i - OBS_i}{OBS_i} \right| \times 100\% \quad (2)$$

$$MPE = \frac{1}{N} \sum \frac{IPRED_i - OBS_i}{OBS_i} \times 100\% \quad (3)$$

1. Hooker AC, Staatz CE, Karlsson MO. Conditional weighted residuals (CWRES): A model diagnostic for the FOCE method. *Pharm Res* (2007) 24(12):2187-97. doi: 10.1007/s11095-007-9361-x. PubMed PMID: WOS:000250722500002.
2. Lindbom L, Ribbing J, Jonsson EN. Perls-speaks-NONMEM (PsN) - a Perl module for NONMEM related programming. *Comput Meth Prog Bio* (2004) 75(2):85-94. doi: 10.1016/j.cmpb.2003.11.003. PubMed PMID: WOS:000222690900001.
3. Comets E, Brendel K, Mentre F. Computing normalised prediction distribution errors to evaluate nonlinear mixed-effect models: The npde add-on package for R. *Comput Meth Prog Bio* (2008) 90(2):154-66. doi: 10.1016/j.cmpb.2007.12.002. PubMed PMID: WOS:000255381700007.
4. Ding JJ, Wang Y, Lin WW, Wang CL, Zhao LM, Li XG, et al. A Population Pharmacokinetic Model of Valproic Acid in Pediatric Patients with Epilepsy: A Non-Linear Pharmacokinetic Model Based on Protein-Binding Saturation. *Clinical Pharmacokinetics* (2015) 54(3):305-17. doi: 10.1007/s40262-014-0212-8. PubMed PMID: WOS:000350103900008.
5. van der Meer AF, Marcus MAE, Touw DJ, Proost JH, Neef C. Optimal Sampling Strategy Development Methodology Using Maximum A Posteriori Bayesian Estimation. *Ther Drug Monit* (2011) 33(2):133-46. doi: 10.1097/FTD.0b013e31820f40f8. PubMed PMID: WOS:000288498100001.
